# Optimal Timing for Ureteral Stent Removal After Ureteroscopy to Minimize Morbidity: A Single‐Center Prospective Comparative Study

**DOI:** 10.1155/aiu/5753359

**Published:** 2026-07-07

**Authors:** Muhammad Abdullah Rahman Shwani

**Affiliations:** ^1^ Kurdistan Higher Council of Medical Specialties, Erbil Teaching Hospital, Erbil, Kurdistan, Iraq

**Keywords:** holmium laser lithotripsy, patient-reported outcomes, quality of life, stent removal timing, ureteral stent, ureteroscopy, USSQ

## Abstract

**Objective:**

To compare patient‐reported morbidity across three ureteral stent removal protocols after ureteroscopy (URS) and holmium laser lithotripsy.

**Patients and Methods:**

We prospectively recruited 180 patients undergoing URS for unilateral ureteric or renal stones (1–1.5 cm) from January 2021 to December 2024 at a single academic center. All procedures were performed by a single surgeon using a semirigid or flexible ureteroscope, holmium laser lithotripsy, and placement of a 6Fr × 26 cm double‐J stent. Patients were sequentially allocated into three groups based on stent removal timing: Group A (24–48 h, *n* = 60), Group B (3–5 days, *n* = 60), and Group C (7–14 days, *n* = 60). The primary outcome was the Ureteral Stent Symptom Questionnaire (USSQ) total score. Secondary outcomes included Visual Analog Scale (VAS) pain score at Day 7, opioid tablet consumption, complication rates (Clavien–Dindo), patient satisfaction (1–10 scale), and days to return to normal activities. Propensity score analysis was performed to adjust for potential confounding.

**Results:**

Baseline characteristics were similar across groups. Group A had the lowest USSQ total score (68.4 ± 12.6 vs. 76.8 ± 13.4 in Group B vs. 89.7 ± 15.2 in Group C; *p* < 0.001). VAS pain score at Day 7 was also lowest in Group A (2.1 ± 1.2 vs. 3.9 ± 1.5 in Group B vs. 4.8 ± 1.8 in Group C; *p* < 0.001). Opioid tablets consumed were fewer in Group A (2.3 ± 2.1 vs. 5.6 ± 2.9 in Group B vs. 8.4 ± 3.6 in Group C; *p* < 0.001). Complication rates did not differ significantly between groups (*p* = 0.42). Patient satisfaction was highest in Group A (8.9 ± 1.1 vs. 7.5 ± 1.4 in Group B vs. 6.2 ± 1.8 in Group C; *p* < 0.001), and return to normal activities was fastest in Group A (3.2 ± 1.4 vs. 5.9 ± 1.9 in Group B vs. 8.7 ± 2.3 days in Group C; *p* < 0.001). On multivariable analysis, longer stent duration was the strongest independent predictor of higher USSQ score (*β* = 0.42, *p* < 0.001), followed by female sex (*β* = 0.18, *p* = 0.02) and age under 50 years (*β* = 0.15, *p* = 0.04).

**Conclusion:**

In this prospective comparative study, stent removal at 24–48 h was associated with lower morbidity, earlier return to activities, and higher patient satisfaction compared to later removal, without an observed increase in complications. These findings suggest that early removal may be beneficial in appropriately selected patients without contraindications. Removal at 3–5 days may represent a practical alternative when earlier removal is not feasible. Adequately powered randomized trials are needed to confirm these findings.

## 1. Introduction

Ureteroscopy (URS) with holmium laser lithotripsy is widely considered the standard treatment for ureteric and renal stones (1–1.5 cm), achieving a stone‐free rate of more than 85% with an acceptable safety profile [1, 2]. After URS, a ureteral stent is commonly placed to maintain drainage and prevent obstruction from postoperative edema or residual fragments [3, 4].

However, stents are often poorly tolerated. Pain, hematuria, dysuria, urinary frequency, and disruption of daily life and sexual function are reported in 60%–80% of patients [5, 6]. Daily symptom profiling from the multicenter STENTS cohort showed that pain and urinary bother peak in the first two postoperative days and persist throughout the indwelling period, with wide variation between patients [7].

The Ureteral Stent Symptom Questionnaire (USSQ), developed and validated by Joshi et al. in 2003, captures this burden across six domains: urinary symptoms, body pain, general health, work performance, sexual function, and additional problems [8]. It remains the most commonly used patient‐reported outcome tool in stent research [9, 10].

The optimal duration of stent placement after uncomplicated URS remains unsettled [11, 12]. The traditional 7–14‐day window was based on the assumption that ureteral edema takes time to resolve [13]. However, this assumption is increasingly challenged. A recent randomized trial found that stent removal at 3 days produced significantly lower urinary and pain scores than removal at 7 days [14], and a large prospective series of 1547 patients reported postprocedural event rates of 15% with Day‐5 removal versus 44.9% with Day‐14 removal [15]. On the other hand, one retrospective review found that complications within 3 days of removal were more common in the early group (23% vs. 3%, *p* = 0.026), raising questions about very short durations [16].

Current EAU guidelines support selective rather than routine stenting after uncomplicated URS but do not recommend a specific removal window [17, 18]. Prolonged stenting drives unplanned emergency visits, extra clinic attendances, and lost productivity—costs that are rarely accounted for in clinical decision‐making [19, 20]. The effect of modern laser techniques, including dusting versus fragmentation, on the optimal stent duration also remains poorly defined [21].

Against this background, four gaps stand out: (1) most studies compare only two time points; (2) few use the USSQ as a primary endpoint; (3) the link between stent duration and opioid use has rarely been examined; and (4) most existing data come from retrospective or small series.

This study was designed to address these gaps by prospectively comparing three removal protocols—24–48 h, 3–5 days, and 7–14 days—using the USSQ as the primary outcome. We hypothesized that earlier removal would reduce morbidity, including USSQ scores, pain, and opioid consumption, without increasing the rate of urinary tract infection, acute kidney injury, or emergency department visits.

## 2. Patients and Methods

### 2.1. Study Design and Setting

We conducted a prospective comparative cohort study at a single academic tertiary urology unit from January 2021 to December 2024. The study was approved by the Institutional Review Board (approval number 2640, December 30, 2020), and all participants provided written informed consent prior to enrollment. The Declaration of Helsinki was followed throughout, and reporting adheres to STROBE guidelines.

### 2.2. Patient Selection

We included adults aged 18 to 75 years with unilateral ureteric or renal stones measuring 1–1.5 cm who were scheduled for URS with holmium laser lithotripsy and had an ASA physical status of I–III.

Patients were excluded if they had bilateral stones requiring simultaneous treatment, a solitary kidney, chronic kidney disease (eGFR < 30 mL/min/1.73 m^2^), active urinary tract infection at the time of surgery, pregnancy or breastfeeding, anatomic abnormalities (horseshoe kidney, ureteral stricture, or ureteropelvic junction obstruction), prior ipsilateral ureteral surgery within 6 months, intraoperative ureteral perforation or significant mucosal injury, residual stone burden > 4 mm necessitating a planned second‐stage procedure, or cognitive impairment precluding questionnaire completion.

### 2.3. Sample Size

Based on published USSQ data [14], we anticipated a mean total score of 90 ± 15 in the 7–14‐day group and 75 ± 15 in the 24–48‐h group (effect size *d* = 1.0). With a two‐sided alpha level of 0.05, 80% power, and a Bonferroni correction for three pairwise comparisons (adjusted alpha = 0.017), the required sample size was 52 patients per group. Allowing for a 15% dropout rate, we enrolled 60 per group (180 analyzed; 190 initially enrolled; 10 were excluded post‐enrollment but before analysis due to 6 lost to follow‐up, 2 withdrew consent, and 2 had incomplete USSQ data).

### 2.4. Group Assignment

Patients were allocated to one of three groups based on stent removal timing: Group A (24–48 h), Group B (3–5 days), or Group C (7–14 days).

Allocation followed a strict chronological sequential protocol: The first 60 consecutive eligible patients were assigned to Group A, the next 60 to Group B, and the final 60 to Group C. No patient preference or surgeon recommendation influenced assignment. This was not a randomized trial.

Outcome assessors reviewing complications by chart audit were blinded to group assignment. However, patients and the treating urologist could not be blinded due to the nature of the intervention. To account for potential confounding due to nonrandomized allocation, a post hoc propensity score analysis was performed.

### 2.5. Surgical Technique

All procedures were performed by a single endourologist with experience of more than 500 URS cases. Patients received general anesthesia with endotracheal intubation. A semirigid ureteroscope (Karl Storz, 8/9.8 Fr) was used for ureteric and lower‐pole renal stones; a single‐use flexible ureteroscope (Hugemed, 7.5 Fr working channel) was used for mid‐ and upper‐pole renal stones. Lithotripsy was performed with a holmium:YAG laser (Lumenis Pulse 120H, 20–30 W); settings were adjusted to stone composition and location (dusting: 0.6–1.0 J at 10–20 Hz; fragmentation: 1.0–1.5 J at 10–15 Hz). Fragments larger than 3 mm were retrieved with a 1.9 Fr nitinol basket; smaller debris was left for spontaneous passage. A 6 Fr double‐J stent (Boston Scientific Urovision or Uromed, length 24–28 cm, adjusted to patient height) was placed under fluoroscopic guidance in all cases. No urethral Foley catheter was placed postoperatively.

### 2.6. Stent Removal

All stents were removed by flexible cystoscopy under local anesthesia (intraurethral lidocaine gel) in the outpatient clinic. Patients received oral ibuprofen 400 mg or acetaminophen 500 mg 30 minutes beforehand.

### 2.7. Postoperative Management

Perioperative antibiotic prophylaxis consisted of intravenous cefazolin 1 g at induction followed by oral ciprofloxacin 500 mg twice daily for 3 days (trimethoprim–sulfamethoxazole for penicillin or fluoroquinolone allergy). Pain was managed with scheduled ibuprofen 400 mg three times daily; oxycodone 5 mg tablets (maximum 20) were prescribed for breakthrough pain. Tamsulosin 0.4 mg daily was offered but not mandated. Patients were advised to maintain fluid intake of at least 2 L per day and to avoid heavy lifting or strenuous exercise until stent removal.

### 2.8. Outcome Measures

Primary outcome: The USSQ total score was administered at the time of stent removal, except for Group A, in whom the stent had already been removed at 24–48 h, so the USSQ was administered on Day 7 to allow a standardized comparison time point across all groups [8]. This means that Group A completed the USSQ without a stent in situ, while Groups B and C completed it with the stent still in place. This difference is an important limitation of the study design.

The USSQ covers six domains: urinary symptoms (11 items, range 11–55), body pain (9 items, range 9–45), general health (6 items, range 6–30), work performance (7 items, range 7–35), sexual matters (4 items, range 4–20), and additional problems (1 item). Total score ranges from 38 to 190; higher scores indicate greater symptom burden.

### 2.9. Secondary Outcomes

VAS pain scores (0–10) at 24 and 48 h, Day 7, and Day 30; total oxycodone tablet consumption recorded by patient diary and pill count; complication rates (culture‐proven UTI defined as > 10^5^ CFU/mL with symptoms; AKI defined as serum creatinine rise ≥ 0.3 mg/dL or ≥ 1.5 × baseline; unplanned ED visits and hospital readmissions within 30 days); patient satisfaction on a 0–10 scale at Day 30; days to return to normal activities; and stent migration assessed by KUB radiograph at removal.

### 2.10. Follow‐Up

Patients were contacted by phone at 24 and 48 h to assess pain and early complications. Group A attended for stent removal at 24–48 h; Groups B and C attended at 3–5 and 7–14 days, respectively. All patients attended a clinic visit at Day 30 for final outcome assessment, diary collection, and complication review. A phone call was made on Day 7 to capture USSQ data for Group A.

### 2.11. Statistical Analysis

Continuous variables are reported as mean ± SD or median (IQR) based on normality assessed by the Shapiro–Wilk test. Categorical variables are reported as frequencies and percentages.

Between‐group comparisons used one‐way ANOVA with Bonferroni‐corrected post hoc tests for continuous outcomes and chi‐square or Fisher’s exact test for categorical outcomes.

#### 2.11.1. Propensity Score Analysis

We constructed a multinomial propensity score model using the following baseline covariates: age, sex, BMI, ASA class, stone size, stone location, operative time, and ureteroscope type. Inverse probability of treatment weighting (IPTW) was applied. Balance was considered adequate when standardized mean differences (SMD) were below 0.10. Primary and secondary outcomes were reanalyzed in the weighted cohort.

#### 2.11.2. Linear Regression (Predictors of USSQ Score)

Independent predictors of USSQ total score were identified by multiple linear regression. To avoid overfitting, given our sample size (*n* = 180), we limited the model to the following prespecified covariates: stent duration (days), age, sex, BMI, stone size, and stone location. Age, sex, BMI, stone size, stone location, operative time, and ureteroscope type were entered as covariates in our study. Operative time and ureteroscope type were not included in the final model due to limited events. Results are reported as standardized beta coefficients (*β*) with 95% confidence intervals.

All tests were two‐sided, and *p* < 0.05 was considered statistically significant. Statistical analyses were performed using SPSS Version 27.0 (IBM Corp., Armonk, NY, USA).

## 3. Results

**FIGURE 1:**
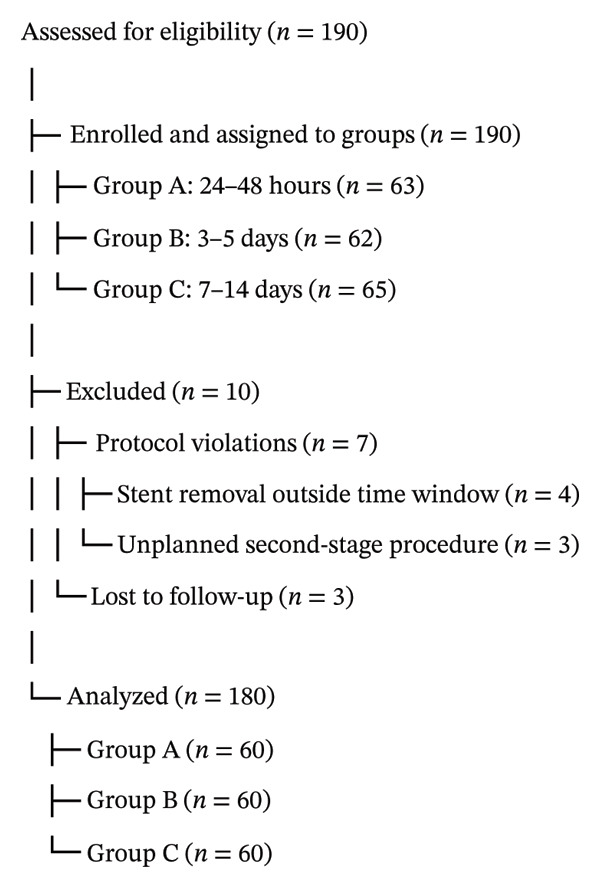
Consort flow diagram.

From January 2021 to December 2024, we enrolled 190 patients. Ten were excluded from the analysis: seven due to protocol violations (four had stent removal outside the designated time window; three needed an unplanned second‐stage procedure for residual stone burden > 4 mm), and three were lost to follow‐up. This left 180 patients for analysis (60 in each group). The patient flow is shown in Figure 1.

The baseline demographic and clinical characteristics are shown in Table 1. Before propensity score adjustment, age, sex, BMI, ASA class, stone size, stone location, operative time, and flexible ureteroscope use showed no significant differences among groups (all *p* > 0.05). After IPTW adjustment, all SMD were < 0.10, indicating good balance.

**TABLE 1:** Baseline demographic and clinical characteristics.

Characteristic	Group A (24–48 h) *n* = 60	Group B (3–5 d) *n* = 60	Group C (7–14 d) *n* = 60	*p* value
Age (years), mean ± SD	48.3 ± 12.4	50.1 ± 13.2	49.7 ± 11.8	0.67
Male sex, *n* (%)	38 (63.3)	36 (60.0)	40 (66.7)	0.72
BMI (kg/m^2^), mean ± SD	27.8 ± 4.2	28.3 ± 4.6	27.5 ± 4.1	0.58
ASA class, *n* (%)				0.81
I	22 (36.7)	20 (33.3)	24 (40.0)	
II	32 (53.3)	34 (56.7)	30 (50.0)	
III	6 (10.0)	6 (10.0)	6 (10.0)	
Stone location, *n* (%)				0.76
Ureteric	34 (56.7)	32 (53.3)	36 (60.0)	
Renal	26 (43.3)	28 (46.7)	24 (40.0)	
Stone size (mm), mean ± SD	11.8 ± 2.4	12.1 ± 2.6	11.6 ± 2.3	0.52
Operative time (min), mean ± SD	52.3 ± 18.6	54.8 ± 20.2	51.7 ± 17.9	0.63
Flexible ureteroscope used, *n* (%)	26 (43.3)	28 (46.7)	24 (40.0)	0.76
Tamsulosin use, *n* (%)	48 (80.0)	46 (76.7)	50 (83.3)	0.64

*Note:* Comparisons were performed using one‐way ANOVA for continuous variables and the chi‐square test for categorical variables.

Abbreviations: ASA, American Society of Anesthesiologists; BMI, body mass index; SD, standard deviation.

### 3.1. Primary Outcome: USSQ Total Score

The total score on the USSQ differed significantly among the three groups (*p* < 0.001, one‐way ANOVA). Group A had the lowest mean score (68.4 ± 12.6), followed by Group B (76.8 ± 13.4) and Group C (89.7 ± 15.2). Pairwise comparisons showed significant differences between Group A and Group C (mean difference −21.3, 95% CI −26.8 to −15.8, *p* < 0.001), Group B and Group C (mean difference −12.9, 95% CI −18.4 to −7.4, *p* = 0.002), and Group A and Group B (mean difference −8.4, 95% CI −13.9 to −2.9, *p* = 0.03). Domain scores are presented in Table 2. For all clinically significant domains, Group A scored the lowest (best) and Group C the highest (worst).

**TABLE 2:** Ureteral Stent Symptom Questionnaire (USSQ) scores.

USSQ domain	Group A (24–48 h) *n* = 60	Group B (3–5 d) *n* = 60	Group C (7–14 d) *n* = 60	*p* value
Urinary symptoms (11–55)	24.6 ± 5.8	28.3 ± 6.2	34.2 ± 7.1	< 0.001
Body pain (9–45)	18.2 ± 4.3	21.6 ± 4.8	26.8 ± 5.6	< 0.001
General health (6–30)	12.4 ± 3.2	13.8 ± 3.6	16.2 ± 4.1	< 0.001
Work performance (7–35)	9.8 ± 2.6	10.4 ± 2.9	9.2 ± 2.4	0.08
Sexual matters (4–20)	3.4 ± 1.7	2.7 ± 1.4	3.3 ± 1.6	0.06
Total scores (38–39)	68.4 ± 12.6	76.8 ± 13.4	89.7 ± 15.2	< 0.001

*Note:* Values are mean ± SD. Higher scores indicate worse symptoms—*p* values from one‐way ANOVA.

### 3.2. Secondary Outcomes

#### 3.2.1. Pain Scores

VAS pain scores are summarized in Table 3. At 24 h, scores were similar across all groups (*p* = 0.52), indicating similar immediate postoperative pain regardless of stent duration. Significant differences emerged at 48 h (*p* = 0.004) and were more pronounced at Day 7 when Group A had a mean VAS of 2.1 ± 1.2, significantly lower than Group B (3.4 ± 1.5, *p* = 0.001) and Group C (4.8 ± 1.8, *p* < 0.001). By Day 30, scores had decreased to low values in all groups and were no longer significantly different (*p* = 0.18).

**TABLE 3:** Visual Analog Scale (VAS) pain scores over time.

Time point	Group A (24–48 h) *n* = 60	Group B (3–5 d) *n* = 60	Group C (7–14 d) *n* = 60	*p* value
24 h	4.2 ± 1.8	4.5 ± 1.9	4.3 ± 1.7	0.52
48 h	3.1 ± 1.4	3.8 ± 1.6	4.1 ± 1.7	0.004
Day 7	2.1 ± 1.2	3.4 ± 1.5	4.8 ± 1.8	< 0.001
Day 30	0.8 ± 0.9	1.1 ± 1.0	1.2 ± 1.1	0.18

*Note:* Values are mean ± SD. VAS scale: 0 = *no pain*, 10 = *worst imaginable pain*. *p* values from one‐way ANOVA.

#### 3.2.2. Opioid Consumption

Total opioid use over 30 days was significantly different among groups (*p* < 0.001). Group A patients took 2.3 ± 2.1 oxycodone 5 mg tablets (total over 30 days), compared to 4.8 ± 2.9 in Group B and 8.4 ± 3.6 in Group C. All pairwise differences were significant (*p* < 0.01 for each comparison), suggesting a dose–response relationship between stent duration and analgesic requirement.

#### 3.2.3. Complications

Thirty‐day complication rates are shown in Table 4. No significant differences were found in rates of urinary tract infection (*p* = 0.68), acute kidney injury (*p* = 0.82), unplanned emergency department visits (*p* = 0.54), or hospital readmission (*p* = 0.62). No patient in any group had urosepsis, ureteral stricture, or stent migration requiring intervention. The overall complication rate was 16.7% in Group A, 20.0% in Group B, and 25.0% in Group C (*p* = 0.42).

**TABLE 4:** Complication rates at 30‐day follow‐up.

Complication	Group A (24–48 h) *n* = 60	Group B (3–5 d) *n* = 60	Group C (7–14 d) *n* = 60	*p* value
Urinary tract infection, *n* (%)	4 (6.7)	3 (5.0)	5 (8.3)	0.68
Acute kidney injury, *n* (%)	1 (1.7)	2 (3.3)	2 (3.3)	0.82
Emergency department visit, *n* (%)	5 (8.3)	7 (11.7)	8 (13.3)	0.54
Hospital readmission, *n* (%)	2 (3.3)	3 (5.0)	4 (6.7)	0.62
Urosepsis, *n* (%)	0 (0)	0 (0)	0 (0)	1.00
Stent migration, *n* (%)	1 (1.7)	0 (0)	1 (1.7)	0.60
Any complication, (*n*%)	10 (16.7)	12 (20.0)	15 (25.0)	0.42

*Note: p* values from the chi‐square test or Fisher’s exact test.

#### 3.2.4. Patient Satisfaction and Return to Activities

Group A patients had higher satisfaction scores (8.9 ± 1.1) compared to Group B (7.4 ± 1.6) and Group C (6.2 ± 1.8) (*p* < 0.001 for both comparisons). Time to return to normal activities was shortest in Group A (3.2 ± 1.4 days), followed by Group B (5.8 ± 2.1 days) and Group C (8.7 ± 2.3 days). All pairwise differences were significant (*p* < 0.001). These data are presented in Table 5.

**TABLE 5:** Patient satisfaction and return to normal activities.

Outcome	Group A (24–48 h) *n* = 60	Group B (3–5 d) *n* = 60	Group C (7–14 d) *n* = 60	*p* value	Outcome
Patient satisfaction (0–10), mean ± SD	8.9 ± 1.1	7.4 ± 1.6	6.2 ± 1.8	< 0.001	Patient satisfaction (0–10), mean ± SD
Return to activities (days), mean ± SD	3.2 ± 1.4	5.8 ± 2.1	8.7 ± 2.3	< 0.001	Return to activities (days), mean ± SD.

*Note:* Satisfaction scale: 0 = *completely dissatisfied*, 10 = *completely satisfied*. *p* values from one‐way ANOVA.

Abbreviation: SD, standard deviation.

#### 3.2.5. Multivariable Analysis

Multivariable linear regression identified independent predictors of a higher USSQ total score. Longer stent duration was the strongest predictor (*β* = 0.42, 95% CI: 0.28–0.56, *p* < 0.001), followed by female sex (*β* = 0.18, 95% CI: 0.04–0.32, *p* = 0.02) and age under 50 years (*β* = 0.15, 95% CI: 0.01–0.29, *p* = 0.04). Stone size, stone location, operative time, BMI, and ureteroscope type were not significant predictors (all *p* > 0.05). The model accounted for 38% of the variance in the USSQ total score (*R* ^2^ = 0.38, *p* < 0.001).

## 4. Discussion

Stent‐related morbidity after URS is not a minor inconvenience—it is the major cause of unplanned postoperative contact, analgesic use, and loss of productivity in what is otherwise a day‐case procedure. However, for some time, the question of how long the stent should remain in place has been surprisingly little examined. Most urologists are trained to remove stents at 1–2 weeks, a tradition rooted in custom rather than evidence. This study examined whether that tradition still holds, and the short answer is that it does not.

Among 180 patients sequentially allocated to stent removal at 24–48 h, 3–5 days, or 7–14 days, those in the earliest removal group had significantly lower USSQ total scores, less pain at Day 7, markedly lower opioid use, higher satisfaction, and quicker return to work and daily activities. However, what made us most confident in considering early removal was not the symptom data—it was the complication data. Infection rates, acute kidney injury, emergency visits, and readmissions were similar across the three groups. This is a key clinical finding, as it addresses the safety argument used to justify longer‐term stenting. Multivariable analysis supported this finding.

Stent duration was the strongest independent predictor of USSQ total score (*β* = 0.42, *p* < 0.001)—stronger than stone size, operative time, or ureteroscope type. Female sex (*β* = 0.18, *p* = 0.02) and age under 50 years (*β* = 0.15, *p* = 0.04) also predicted worse symptom scores, consistent with previous studies reporting that these groups are particularly sensitive to indwelling stents [7, 22]. Collectively, these data support the hypothesis that the stent is a major contributor to morbidity, and earlier removal may reduce it.

The most comparable randomized trial to ours is that of Heidenberg et al., who compared 3‐day versus 7‐day stent removal in 100 patients [14]. They found significantly lower urinary and pain scores in the shorter‐duration arm, both while the stent was in situ and after removal. Our Group A (24–48‐h removal) had a mean USSQ total score of 68.4 ± 12.6, which was lower than the 3‐day group in their series, suggesting that moving the removal window even earlier provides incremental benefit. We believe this incremental benefit is worth the logistical effort of arranging removal within 48 h of surgery, particularly given the opioid and recovery data.

The largest dataset in this area comes from Banerjee et al., who prospectively recruited 1547 patients and reported 30‐day postprocedure events in 15% of the Day‐5 removal group compared to 44.9% of the Day‐14 removal group [15]. Our complication rates (16.7%–25.0%) were lower than those reported by Banerjee et al., primarily due to our strict exclusion criteria—patients with anatomic abnormalities, ureteral perforation, or residual stone burden > 4 mm were excluded. This is not a weakness of our study; rather, it reflects a conscious choice to select the population for whom early removal is most appropriate.

Paul et al., in a retrospective review of 247 URS patients, reported that events occurring within 3 days of stent removal were more common in the 3‐day removal group than in the 7‐day group (23% vs. 3%, *p* = 0.026) [16]. We do not dismiss this finding. It serves as a reminder that early removal is not for everyone, and our exclusion criteria—particularly regarding intraoperative complications and residual fragments—are not merely administrative. They are the reason our early removal group was safe.

The FAST 3 trial by Reicherz et al. tested transient mono‐J drainage for 6 h against traditional double‐J stenting for 3–5 days [23]. That trial was stopped early because the mono‐J arm had a numerically higher reintervention rate (35.5% vs. 16.7%), although the difference was not significant (*p* = 0.27). We interpret this result as indicating that 6 h of external drainage may be insufficient, whereas 24–48 h of internal double‐J stenting appear to provide adequate protection for ureteral function while minimizing morbidity.

Two Chinese randomized trials examined the other end of the spectrum (2‐week vs. 4‐week) after holmium laser lithotripsy. Both showed significantly increased urinary irritative symptoms, infection, flank pain, and encrustation with longer duration [24, 25]. These findings are not surprising, but they help define the upper bound of the duration spectrum and confirm that the direction of travel is correct: less stenting duration is beneficial. The question is where to stop, and our data suggest 24–48 h for appropriately selected patients.

Porto et al. pooled four studies to compare removal at ≤ 5 days versus ≥ 6 days and found no overall difference in postprocedure events (OR 1.26, 95% CI 0.22–7.25, *p* = 0.79) but with high heterogeneity (I^2^ = 98%) [26]. The pattern of complications varied by timing—flank pain and hematuria were more common with longer stenting, while fever and dysuria were less common with early stenting. We interpret this heterogeneity as reflecting genuine variation in patient populations and surgical contexts rather than a deficiency in the evidence base, and it is why blanket protocols are unlikely to be as effective as individualized treatment.

The STENTS cohort study identified daily symptom trajectories in 40 patients and showed that pain and urinary bother peak in the first 48 postoperative hours and persist as long as the stent remains in place [7]. This temporal pattern offers a physiological explanation for our findings. The stent does not become more comfortable with time—it remains uncomfortable. Removing it at 24–48 h interrupts the symptom trajectory near its peak.

Regarding stentless URS: Byrne et al. and Borboroglu et al. have both shown in randomized trials that routine stenting is not necessary after uncomplicated URS, and selective omission is safe [27, 28]. We did not include a stentless arm, partly because our stones were 1–1.5 cm—a size for which most endourologists prefer to stent—and partly because our research question concerned timing, not the decision to stent. Our results do not contradict the stentless literature; rather, both lines of evidence agree that less stent exposure means less morbidity.

The mechanisms of stent‐induced morbidity are well described: bladder irritation from the intravesical coil, ureteral spasm, vesicoureteral reflux, and trigonal stimulation [5, 6]. In our sample, urinary symptoms and body pain were the USSQ domains that differed significantly among the three groups—the domains most closely associated with these mechanisms. Notably, pain scores were consistently low on Day 30 across all three groups, regardless of when the stent was removed. This observation suggests that the stent itself, not the surgical trauma, is the primary cause of morbidity. Once the stent is removed, the patient recovers quickly.

The independent effect of female sex on USSQ scores deserves brief comment. Inoue et al. reported in a randomized trial of string versus cystoscopic stent removal that the pain benefit from string removal was significant in men but not women [29]. This suggests that the female experience of indwelling stents differs and may not be easily modified by removal technique alone. Shorter urethral length, higher baseline rates of overactive bladder, and pelvic floor differences may also contribute [22]. In practice, this means female patients might benefit most from early removal, and this factor should be addressed in preoperative counseling.

Our finding that age under 50 years predicted worse USSQ scores is consistent with Asdemir et al., who reported that younger patients were significantly less tolerant of indwelling stents [30]. They also noted that some patients had such mild symptoms that they neglected or forgot stent removal entirely, highlighting the importance of clear postoperative instructions and reminder systems [30].

We wish to highlight our opioid data, as this outcome has been understudied in the stent literature. Group A patients used a mean of 2.3 oxycodone 5 mg tablets over 30 days, whereas Group C used 8.4 tablets—a 73% difference. In an era when reducing opioid prescribing is a clinical and public health priority, a protocol change that reduces opioid use by nearly three‐quarters over 1 month, without any increase in complications, represents a meaningful improvement. This is a compelling justification for a change in practice.

Recovery data show a similar pattern. Group A returned to normal activities in 3.2 days; Group C took 8.7 days, a difference of 5.5 days of lost productivity per patient. Multiplied by the hundreds of thousands of ureteroscopies performed annually, the population‐level economic impact is substantial. We have not conducted a cost‐effectiveness analysis, so we cannot provide a precise estimate, but the direction of effect is clear.

Our results support 24–48‐h stent removal for patients similar to those we enrolled: adults with unilateral stones of 1–1.5 cm, an uncomplicated intraoperative course, no ureteral perforation or significant mucosal injury, and residual stone burden of 4 mm or less. Patients with a solitary kidney, chronic kidney disease, anatomic abnormalities, or significant intraoperative complications were excluded, and our findings cannot be extrapolated to these populations.

In practice, early removal requires several elements beyond surgical judgment. First, patients need clear instructions about symptoms (fever, severe pain, inability to void) that should prompt urgent contact before the scheduled removal appointment. The clinic must be able to accommodate removal within 48 hours—a requirement feasible in most outpatient urology settings. A postoperative phone call at 24 and 48 h provides reassurance and a safety net. All stents in this series were removed by flexible cystoscopy under local anesthesia in the outpatient clinic, without operating room time. Finally, patient preference matters. Some patients may be more comfortable waiting a few days, and this is a reasonable choice to respect.

## 5. Limitations

The nonrandomized, sequential allocation design is the most significant limitation. Although we performed propensity score analysis with IPTW to adjust for measured confounders, unmeasured differences between groups may still exist. The single‐surgeon, single‐center design limits generalizability. A surgeon using different techniques or a center with a different case mix might observe different results. We did not include a stentless arm, so we cannot determine whether the lowest‐risk patients required a stent at all. Alpha‐blockers were offered but not mandated, representing a potential confounding variable, as they may independently reduce stent‐related symptoms. A 30‐day follow‐up is adequate for acute morbidity but cannot address long‐term outcomes such as ureteral stricture—although stricture after uncomplicated URS is rare (< 1%) [31]. We did not perform a cost analysis, so our findings on economic impact remain estimates rather than definitive calculations. Additionally, as noted in the Methods, the USSQ was measured after Group A no longer had a stent in situ, whereas Groups B and C still had stents. This timing difference confounds interpretation, as the observed differences may reflect stent presence or absence at the time of measurement rather than the cumulative effect of stent duration [8]. Finally, this study was not powered to detect differences in rare safety outcomes such as ureteral stricture, urosepsis, or stent migration. The absence of such events in our cohort should not be interpreted as evidence of safety.

## 6. Conclusion

In this hypothesis‐generating prospective comparative study of 180 patients undergoing URS, stent removal at 24–48 h was associated with lower USSQ scores, reduced pain, and decreased opioid use compared to removal at 3–5 days or 7–14 days, with no observed difference in complication rates. The 3–5‐day group outperformed the 7–14‐day group across all morbidity measures. Given these findings, early removal (24–48 h) may be considered in patients without a solitary kidney, chronic kidney disease, anatomic abnormalities, or intraoperative complications. Removal at 3–5 days represents a reasonable alternative when earlier removal is not feasible. Future randomized controlled trials are needed to validate these findings in larger and more diverse patient populations. The data obtained and analyzed in the present study are available from the corresponding author upon reasonable request, subject to institutional data‐sharing protocols and patient privacy protections.

## Funding

This research has no specific grant from any government, commercial, or not‐for‐profit funding agency.

## Conflicts of Interest

The authors declare no conflicts of interest.

## Data Availability

The datasets generated and/or analyzed during the current study are not publicly available due to ethical restrictions and patient confidentiality. Still, they are available from the corresponding author on reasonable request.
